# Electrospun P3HT/PVDF-HFP semiconductive nanofibers for triboelectric nanogenerators

**DOI:** 10.1038/s41598-022-19306-1

**Published:** 2022-09-01

**Authors:** Meng-Fang Lin, Kang-Wei Chang, Chia-Hsien Lee, Xin-Xian Wu, Yu-Ching Huang

**Affiliations:** Department of Materials Engineering, Ming Chi University of Technology, New Taipei City, Taiwan

**Keywords:** Devices for energy harvesting, Materials science

## Abstract

This paper describes a simple electrospinning approach for fabricating poly(3-hexylthiophene) (P3HT)/poly(vinylidene fluoride-co-hexafluoropropylene) (PVDF-HFP) semiconductive nanofiber mat triboelectric nanogenerators (TENGs). Measurements of the electrical properties of the P3HT/PVDF-HFP semiconductive nanofiber TENGs revealed that the output voltage could be enhanced up to 78 V with an output current of 7 μA. The output power of the device reached 0.55 mW, sufficient to power 500 red light-emitting diodes instantaneously, as well as a digital watch. The P3HT/PVDF-HFP semiconductive nanofiber TENG could be used not only as a self-powered device but also as a sensor for monitoring human action. Furthermore, it displayed good durability when subjected to 20,000 cycles of an external force test.

## Introduction

The rapidly expanding market for personal electronics—especially wearable electronics and devices for health and environmental monitoring—is increasing the demand for portable power sources^1^. In light of any potentially emerging energy crisis, it will be necessary to search for ways to minimize electronic waste, in particular that originating from the production and disposal of batteries. Alternative energy technologies, including solar cells^2^, thermoelectricity^3,4^, and nanogenerators^5^, are being investigated to provide the electricity for portable personal electronics^6^. The nanogenerator developed by the Wang group^7^ is a promising and attractive means of providing energy for portable devices and, at the same time, minimizing concerns regarding the disposal of batteries and other external power sources. In general, triboelectric nanogenerators (TENGs) convert waste mechanical energy from various sources into electricity; they have attracted much attention for their high energy conversion efficiency and inexpensive fabrication. Most of the materials that have been used in TENGs stem from organic matter, such that their production can readily be expanded to large scale, with prospective industrial applications.

The working principle of a TENG involves the combined effects of triboelectrification and electrostatic induction during the contact of (or friction between) two dielectric materials having opposite triboelectric polarities. Because triboelectrification is a surface charging effect, the structures and compositions of the surfaces of triboelectric materials have critical effects on the output of TENGs. Surface modification (e.g., controlling the surface morphology^8–10^ or introducing charged ions^11–13^) can increase the surface charge density by enlarging the surface area or the difference in triboelectric polarity of the layers. Alternatively, increasing the dielectric constant can enhance the capacitance of the dielectric layer, thereby increasing the surface charge density. Thus, the dielectric constant of a triboelectric material is another important factor affecting the triboelectric performance^14^.

Although virtually all materials exhibit triboelectricity, the development of new triboelectric materials having special micro- and nanostructures can improve the output of TENGs^15,16^. Several types of materials, including insulating polymers [e.g., polytetrafluoroethylene (PTFE), nylon, polydimethylsiloxane (PDMS)]^17,18^, inorganic semiconductors (e.g., TiO_2_, ZnO)^19,20^, conductive polymers [e.g., polypyrrole (PPy), polyaniline (PANI)]^13,21,22^, and metals (e.g., Au, Al)^23,24^, have been used as triboelectric materials in TENGs. Although a TENG displaying improved performance was obtained when using chemically modified TiO_2_ inorganic semiconductor nanomaterials^20^, high temperatures are required for the fabrication of TiO_2_ nanomaterials. Wang et al*.* prepared a TENG incorporating the conducting polymer PPy^21^, but their approach required electrochemical polymerization with anodic aluminium oxide (AAO) as the template, making the fabrication process time-consuming and costly. The electrospinning technique has been largely utilized to construct fiber-structured nanogenerators. The fabrication of electrospun ion gel nanofibers has been reported for flexible triboelectric nanogenerator^25^. Jiang et al., reported the introduction of MXene nanosheet to fabricate an all eletrospun TENG^26^. The output power of both reported devices is not sufficient to light more than 50 light-emitting diodes for practical application. Furthermore, an electrospun of PVDF nanofiber based TENG has been fabricated as a wearable triboelectric nanogenerator. The output power was sufficient to light on 250 LEDs^27^. However, there is lack more practical application.

When the volumetric fraction of conductive fillers approaches the percolation threshold, admixtures of conductive materials can dramatically increase the dielectric permittivity of polymers, while at the same time preserving the mechanical flexibility of the polymer, due to the relatively low filler loading^28,29^. The conductive material of choice for this study was the organic semiconductor polymer poly(3-hexylthiophene) (P3HT), which has attracted much attention for its potential applications in solar cells^30,31^ and transistors^32,33^—the result of its high environmental and thermal stability, high electrical conductivity, and high solution processability^34^. Here, electrospinning was used to blend P3HT with poly(vinylidene fluoride–*co*–hexafluoropropylene) (PVDF-HFP) to produce nanofibers. The electrospun nanofiber mats were then used to fabricate the TENG. To the best of our knowledge, this paper is the first to report a nanofiber-based TENG fabricated from a simple blend of the organic semiconductive polymer P3HT, used to enhance the electrical output properties of the device. The electrical properties of the P3HT/PVDF-HFP nanofibers were superior to those of the pristine PVDF-HFP, resulting from an increase in dielectric constant upon adding the semiconductive P3HT. The maximum output voltage of the P3HT/PVDF-HFP nanofiber TENG device reached up to 78 V with a corresponding output current of 7 μA under a cyclic compressive force of 30 N applied at a frequency of 5 Hz. The maximum output power that could be obtained was 0.55 mW, sufficient to power 500 red light-emitting diodes (LEDs) instantaneously. Furthermore, the device could effectively generate power under various external resistances. As a proof of practical applicability, the semiconductive nanofiber mats were used to power a digital watch, suggesting the possibility of developing a wide range of wearable electronics and self-powered human interactive systems.

## Methods

### Materials

Regioregular p-type P3HT [poly(3-hexylthiophene-2,5-diyl)] was acquired from Sigma–Aldrich and used as received. PVDF-HFP (average molecular weight: 400,000) and tetrahydrofuran (THF) were also purchased from Sigma–Aldrich.

### Preparation

Electrospinning was used to fabricate PVDF-HFP nanofibers as well as their composites with P3HT. A concentration of 17 wt% PVDF-HFP in the THF solution was used for electrospinning to produce the nanofibers. The composite solution of P3HT/PVDF-HFP in the THF solution was prepared by continuous stirring of a mixture of 3 wt% P3HT with PVDF-HFP/THF. The composite solution of P3HT/PVDF-HFP was electrospun using an 18G blunt needle, at a voltage of 15 kV, a pump rate of 0.3 mL/h, and a needle-to-collector distance of 130 mm, to form the nanofibers. All nanofibers were collected over aluminium foil used as a ground surface.

### Material characterization

Scanning electron microscopy (SEM, Hitachi, Model S-5200) was performed at 8 kV to determine the morphologies of the nanofibers and the deposited Ag layer. UV–Vis absorption spectroscopy was performed using a Jasco V-650 spectrometer. Raman spectra were recorded using a Horiba HR 550 spectrometer, with an excitation wavelength of 532 nm. The dielectric constant was measured using a M6632 apparatus. The transferred charge was measured using a Keithley 6517B system electrometer. The surface potential of fabricated nanofiber mats was measured using an electrostatic voltmeter (Dong Il Technology, Model ARM-S050). The voltage and current output from the triboelectric device were measured using an oscilloscope (Tektronix, Model DPO 3040). The dynamic mechanical pressure was applied by a magnetic shaker (Sinocera, Model JZK-20) under various forces (1–40 N) and frequencies (1–10 Hz). The surface charge was measured using a Keithley 6517B system electrometer (impedance: > 200 TΩ).

## Results and discussion

**Figure 1:**
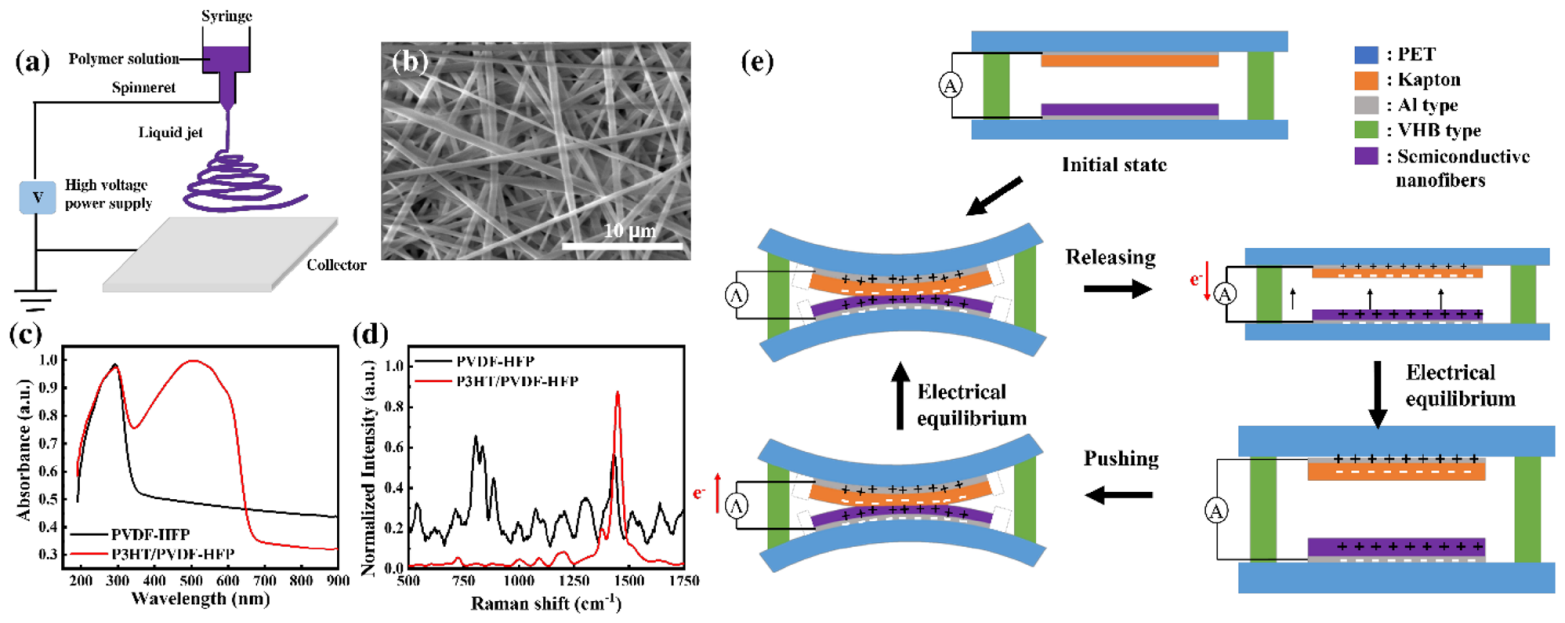
(**a**) Schematic representation of the electrospinning set-up for producing the nanofibers. (**b**) SEM images and (**c**) UV–Vis and (**d**) Raman spectra of electrospun nanofibers composed of pristine PVDF-HFP and 3 wt% P3HT/PVDF-HFP. (**e**) Simplified schematic representation of the electric power generation process of semiconductive nanofiber–based TENGs.

A conventional electrospinning technique was employed to fabricate semiconductive nanofiber mats having thicknesses ranging from 10 to 200 μm (Fig. 1a). The material characteristics of the new composite material were investigated. Figure 1b presents an SEM image of an electrospun P3HT/PVDF-HFP nanofiber. Smooth and bead-free solid nanofibers were arranged in a net with randomly oriented fibers. The average diameter of the nanofibers was approximately 500 nm. To investigate the impact of the conductive P3HT polymer on the electronic properties of the electrospun nanofiber mats, UV–Vis spectra were recorded in the wavelength range 190–900 nm (Fig. 1c). The absorption peak located at 290 nm represented semicrystalline PVDF-HFP^35^. The spectrum of the electrospun P3HT/PVDF-HFP nanofibers featured another absorption peak near 520 nm, corresponding to π–π* intraband transitions in disordered single polymer chains^36,37^. The Raman spectrum of the pristine PVDF-HFP fiber film (Fig. 1d) revealed peaks corresponding to its α-phase at 712 and 887 cm^–1^ and β-phase at 807 cm^–1^^38^. In contrast, the Raman spectrum of the P3HT/PVDF-HFP nanofiber film was dominated by signals for C=C symmetric stretching at 1450 cm^–1^ and C–C intra-ring stretching at 1375 cm^–1^, consistent with π-electron delocalization and a degree of structural order in polythiophenes^39^. The significant changes in both the absorption and Raman spectra upon the incorporation of P3HT hinted at a corresponding change in the dielectric constant.

Figure 1e provides an overview of the working principle of a TENG in vertical contact–separation mode, involving a combination of triboelectrification and electrostatic induction. Prior to contact, no electron flow exists in the initial state in the external circuit. Once an external force is applied to bring the electrospun semiconductive P3HT/PVDF-HFP nanofibers and Kapton into contact, surface charge transfer occurs at the interface, due to a triboelectric effect. The direction of the charge transfer is determined by the relative triboelectric polarity of the two layers. Because Kapton has a strong negative triboelectric polarity^40^, the triboelectric series implies that positive charges are induced at the P3HT/PVDF-HFP nanofibers and negative charges at the Kapton surface. When the external force is switched off, the P3HT/PVDF-HFP and Kapton surfaces are separated to form the released state. At this stage, the separation of the surface charges leads to an increasingly strong dipole moment and creates an electric potential difference between the electrodes. As a consequence, electrons begin to flow from negative to positive potential, and charges accumulate on the electrodes, resulting in a positive electrical signal. Several physical properties of triboelectric materials—in particular, their surface roughness, electron affinity, friction, and capacitance—affect the performance of TENGs. Among them, high capacitance is the most important property for improving the output performance of the devices, measured in terms of their output voltages and currents^40,41^.

**Figure 2:**
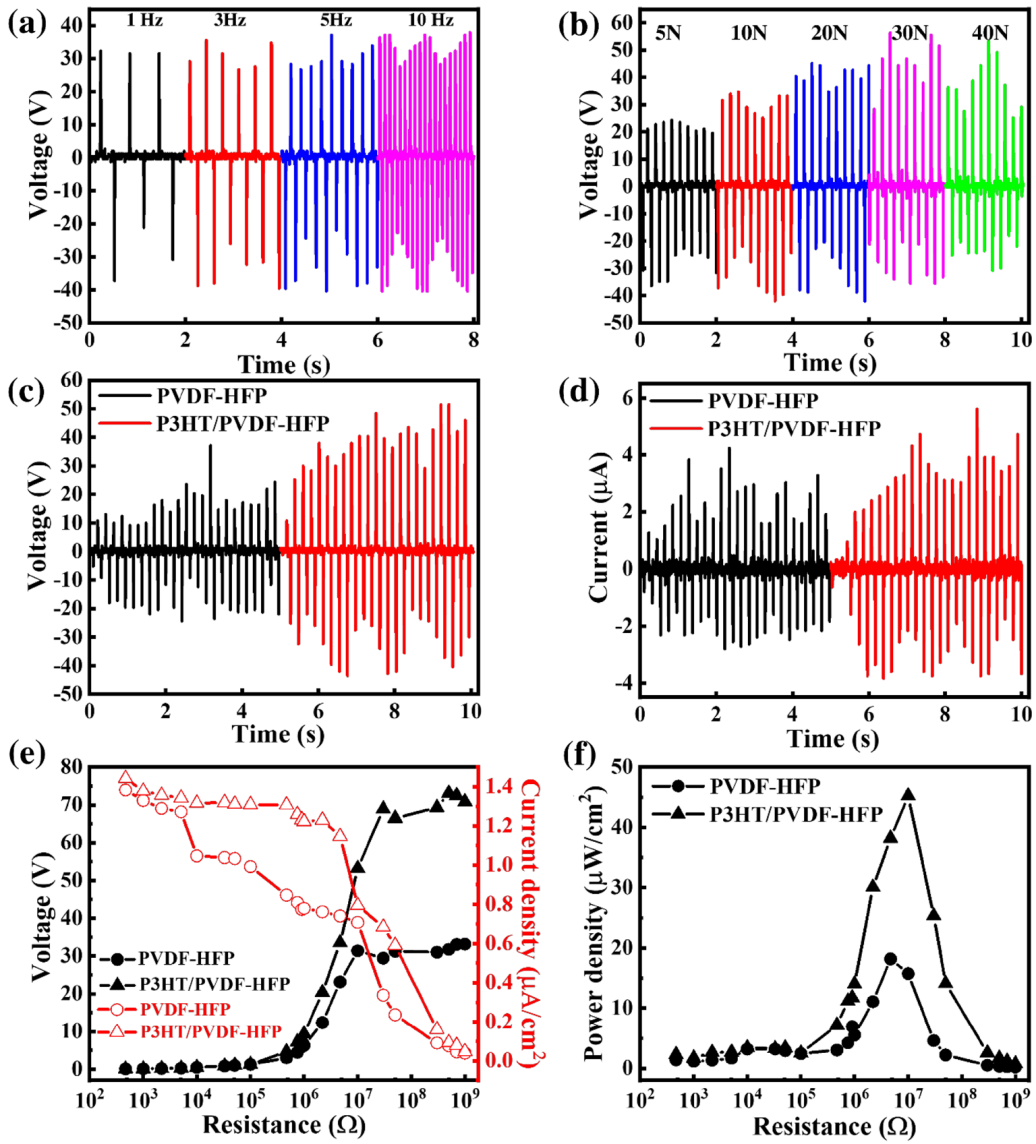
(**a**, **b**) Electrical output voltages of the P3HT/PVDF-HFP nanofibers at various (**a**) frequencies and (**b**) mechanical forces. (**c**) Maximum output voltages and (**d**) currents of TENGs composed of electrospun PVDF-HFP and P3HT/PVDF-HFP nanofibers, measured at 30 N and 5 Hz. (**e**) Output voltage and current density and (**f**) power density of the TENGs composed of PVDF-HFP and P3HT/PVDF-HFP nanofibers, plotted with respect to resistance.

The electrical properties of the P3HT/PVDF-HFP nanofiber were measured while varying the operating frequency, applied external force, and external load. Figure 2a, b display the electrical output voltages of P3HT/PVDF-HFP at various frequencies and mechanical forces, respectively. When the frequency was varied from 1 to 5 Hz under the same applied force of 10 N, the peak output voltage for the P3HT/PVDF-HFP nanofiber mats increased from 52 to 78 V (Fig. 2a). Increasing the operating frequency resulted in more intense friction, which generated more charges, due to faster rate of contact between the P3HT/PVDF-HFP nanofiber mats and the Kapton layer. Nevertheless, the output voltage became unstable at frequencies greater than 10 Hz. This instability arose because, at these high frequencies, the contact and separation processes of the triboelectric layers were incomplete, preventing the surface charge from reaching its maximum value. With a fixed operating frequency of 5 Hz, the output voltage of P3HT/PVDF-HFP nanofiber mats increases from 43 to 78 V upon increasing the applied force from 10 to 30 N. This behaviour presumably resulted from an increased compressive force, leading to significantly improved contact between the triboelectric layers, thereby resulting in the generation of more electric charges. When the applied force was increased to 40 N, the surface of P3HT/PVDF-HFP nanofiber mats was slightly damaged which result in the output voltage drop rather. Figure S1 (Supplementary Information) provides the electrical output currents of the P3HT/PVDF-HFP nanofibers measured at various frequencies and mechanical forces. Figure 2c displays the maximum output performance data of the electrospun PVDF-HFP and P3HT/PVDF-HFP nanofibers having a contact area of 6.25 cm^2^ under a cycled compressive force of 30 N at an applied frequency of 5 Hz. The output voltages of the pristine PVDF-HFP and P3HT/PVDF-HFP nanofiber mats were 41 and 78 V, respectively, under the same mechanical force. The average peak to peak output current of the P3HT/PVDF-HFP nanofiber mats reached up to 7 μA—a value 1.6 times higher than that of the pristine PVDF-HFP nanofiber mats (Fig. 2d)^42^. The combination of the open-circuit voltage and short current led to the maximal power of the P3HT/PVDF-HFP nanofibers (0.55 mW) being greater than that achievable for the PVDF-HFP nanofibers alone (0.18 mW). Furthermore, Fig. 2e, f present the measured voltage outputs, current outputs, and power densities generated by the nanofiber mats under various external load resistances (470–1000 MΩ) when operated at 5 Hz and 30 N. When the loading resistance was below ~ 10 MΩ, the output current density remained at 1.1 μA/cm^2^. The output voltage started to increase as the load resistance increased above ~ 1 MΩ for the P3HT/PVDF-HFP nanofiber mat. Clearly, in contrast to the output current, the output voltage increases with the increase of the resistance until saturation. According to the principle of impedance matching, when the resistance of the external load is equal to the internal resistance of the power supply (namely internal resistance of the TENG), the output power reaches the maximum value. Consequently, the P3HT/PVDF-HFP nanofiber mat TENG exhibited a maximum output power density of 45 μW/cm^2^ at a resistance of 10 MΩ. Compared with the pristine PVDF-HFP nanofiber mat TENG, the power density of the P3HT/PVDF-HFP nanofiber mat TENG was 2.8 times higher, due to its higher surface charge. The electrical output of P3HT/PVDF-HFP TENG is increased by increasing the ratio of P3TH. However, nanofibers with an amount of P3HT beyond 3 wt% cannot be formed in particular due to high conductivity. The high conductivity of the solution can cause large instabilities during the electrospinning process as a high voltage operation (15 kV) is required to fabricate the P3HT/PVDF-HFP nanofiber mat.

**Figure 3:**
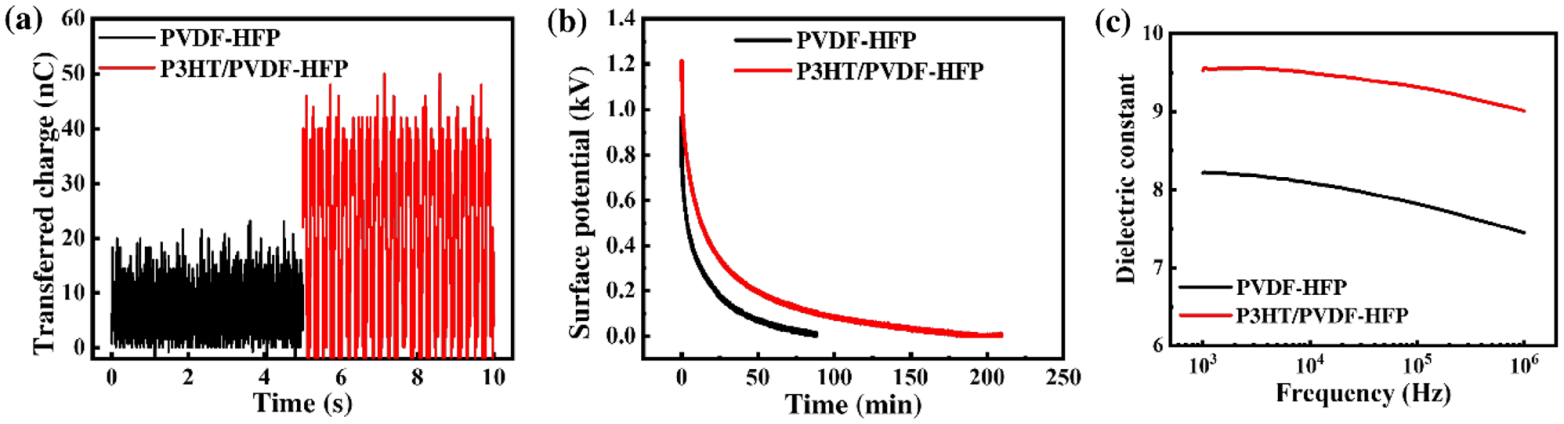
(**a**) Transferred charges and (**b**) retention times of the surface potential for PVDF-HFP and P3HT/PVDF-HFP nanofiber mats after contact friction with the Kapton film. (**c**) Dielectric constants of the PVDF-HFP and P3HT/PVDF-HFP nanofiber mats.

Next, the role of the P3HT polymer in enhancing the output performance of the nanofiber TENG was investigated. Here, the surface charge of the nanofiber mats was measured to provide direct evidence of the effect of P3HT. The measurement was performed using fabricated nanofiber mats after contact friction with the Kapton film. Figure 3a reveals that the surface charge of the P3HT/PVDF-HFP nanofibers was higher than that of the pristine PVDF-HFP nanofibers, suggesting improved capture and storage of the triboelectric electrons after adding P3HT, thereby promoting the corresponding outputs. The initial surface potentials of the pristine PVDF-HFP and P3HT/PVDF-HFP nanofiber mats were 0.9 and 1.9 kV, respectively (Fig. 3b), indicating that the surface charge increased after adding the P3HT polymer, resulting in enhanced electrical output. The surface potential could last for over 200 min with sufficient friction due to enhancing capture and storage of the triboelectric electrons after adding the P3HT polymer. Therefore, the P3HT polymer played an important role in enhancing the frictional surface potential. Furthermore, the dielectric constant of P3HT/PVDF-HFP was higher than that of PVDF-HFP (Fig. 3c). The enhanced dielectric properties presumably resulted from interfacial polarization at the semiconductor–insulator interface, with consideration of the micro-capacitor model and percolation threshold theory, when incorporating the semiconducting-phase P3HT polymer into the PVDF-HFP polymer^28,43,44^. In summary, the dramatic increase in the electrical output of the P3HT/PVDF-HFP TENG over the PVDF-HFP TENG arose from enhancements in the surface charge and potential and from improvements in the dielectric constant after adding the P3HT polymer.

**Figure 4:**
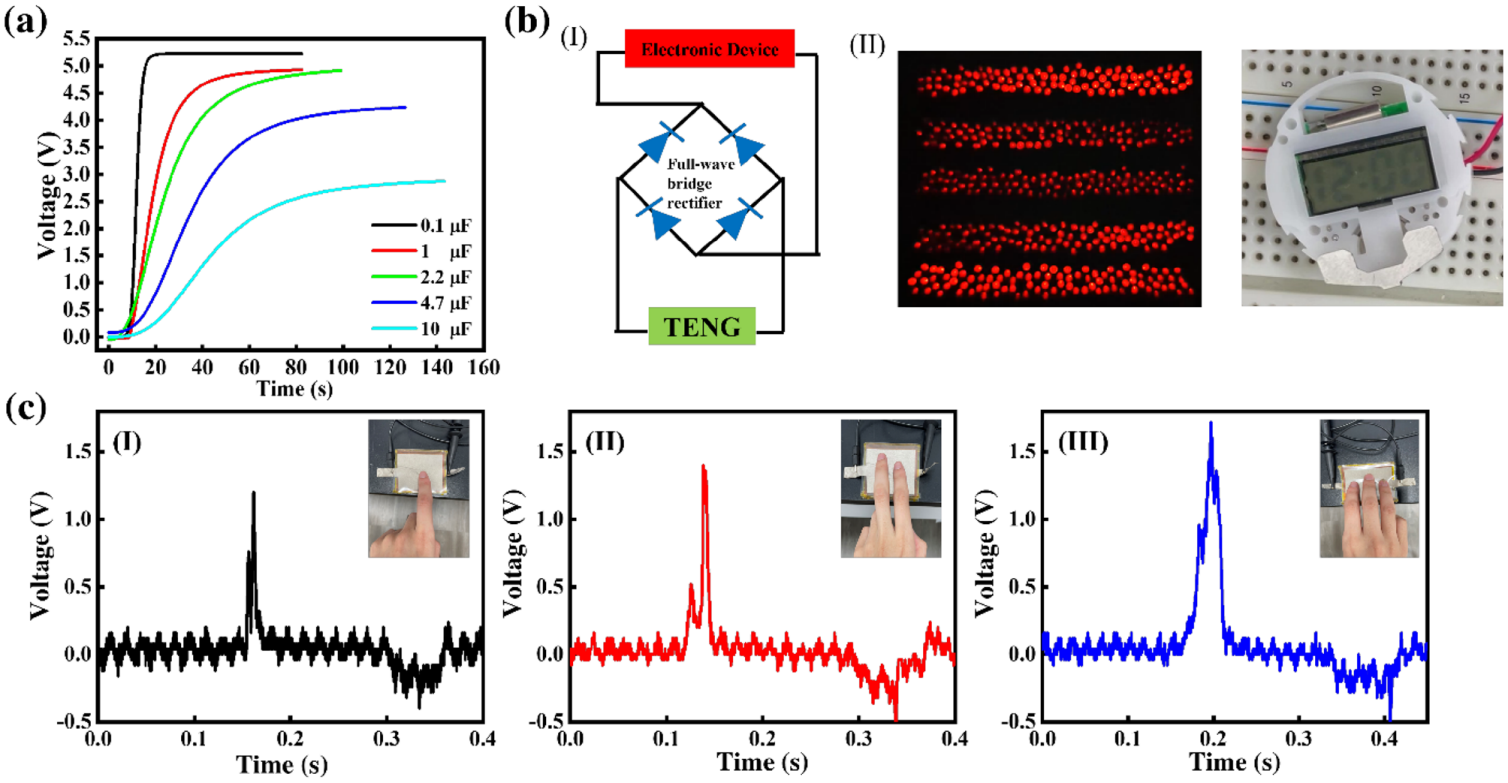
(**a**) Capacitor charging of P3HT/PVDF-HFP nanofibers TENGs having various capacitances. (**b**) (I) Schematic representation of the operating circuit for LED bulbs with a full-wave bridge rectifier and (II) photograph of 500 serially connected LEDs and a digital watch powered by a TENG formed from P3HT/PVDF-HFP nanofiber mats (device size: 2.5 cm × 2.5 cm). (**c**) Sensitivity of the P3HT/PVDF-HFP nanofiber TENG, measured by varying the number of fingers pressing on the device.

Figure 4a displays the voltage curves obtained when charging capacitors of varying capacitance (0.1, 1, 2.2, 4.7, and 10 μF) with a P3HT/PVDF-HFP nanofiber TENG, with the 0.1-μF capacitor undergoing instant charging to 5 V within 15 s. The charging time when using the PVDF-HFP nanofiber mat TENG (25 s) was longer than that for the P3TH/PVDF-HFP nanofiber mat TENG (Fig. S3). Notably, an LED bulb array and a digital watch could be powered by the TENG containing the P3HT/PVDF-HFP nanofiber mats (Fig. 4b and Movie S1). Furthermore, the P3HT/PVDF-HFP TENG device could be used not only as a self-powering device but also as a sensor. The sensitivity of the P3HT/PVDF-HFP TENG was assessed by varying the number of fingers pressing on the device (Fig. 4c). Interestingly, the shape of the output signal, namely the number of peaks, changed depending on the number of fingers pressing on the device. The number of peaks corresponding to each event of pressing the device is due to the asynchronous contact. The time difference between individual peaks is < 0.1 s, which is relatively hard to achieve by repeatedly tapping with a single finger under normal circumstances. Therefore, the number correlated peaks can be considered as an indicator for the number of fingers pressing the device which is not related to the pressure applied on the TENG. Accordingly, this P3HT/PVDF-HFP nanofibers TENG device has potential for application in monitoring human actions and as a power source.

**Figure 5:**
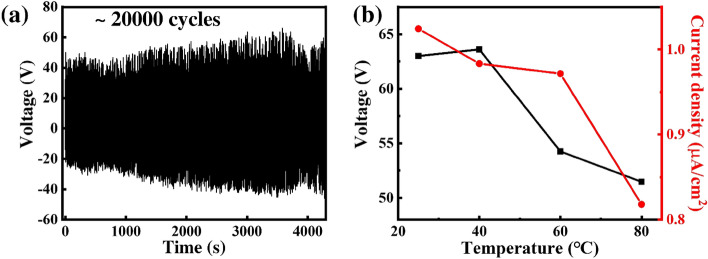
Electrical outputs during (**a**) long-term cycling and (**b**) heating of the P3HT/PVDF-HFP nanofiber TENG.

Durability and stability are extremely important for practical applications of any TENG. To examine the mechanical stability of the device, the electrical output of the P3HT/PVDF-HFP nanofiber TENG was monitored over a duration of 20,000 cycles at 5 Hz (Fig. 5a). The voltage rising slightly within the first 3500 s could be that a longer operation time resulted in accumulating more charge. There was no measurable degradation in the output voltage over 20,000 cycles, suggesting perfect durability and stability for the P3HT/PVDF-HFP nanofiber TENG. Furthermore, a test of the thermal stability of the P3HT/PVDF-HFP nanofiber TENG (Fig. 5b) revealed that its output current density was stable for temperatures of up to 60 °C. At higher temperatures, the output voltage began to fluctuate.

## Conclusions

Semiconductive nanofibers consisting of P3HT/PVDF-HFP have been produced through electrospinning and used as triboelectric materials in TENGs. The electrical output of the PVDF-HFP nanofiber mat TENG improved after incorporating the semiconductive P3HT polymer, due to enhancements in the surface charge, surface potential, and dielectric constant. The optimized fabricated TENG exhibited output powers of up to 0.55 mW, sufficient to operate 500 red LEDs instantaneously. Furthermore, the device could generate power effectively under various external resistance loads. The output voltage of the TENG was stable during long-term cycling and when measured at temperatures of up to 60 °C. As a final demonstration of the potential for application in a wide range of wearable electronics and self-powered human interface systems, a digital watch was powered by the semiconductive nanofiber mat–based TENG in this study.

## Supplementary Information

Supplementary Information 1.

Supplementary Video 1.

## Data Availability

The datasets generated and/or analyzed during the current study are not publicly available due Intellectual Property issues but are available from the corresponding author on reasonable request.
