# Supplementary material for: Electrospun P3HT/PVDF-HFP semiconductive nanofibers for triboelectric nanogenerators
Source: Sci Rep. 2022 Sep 1;12:14842. doi: 10.1038/s41598-022-19306-1 (PMC9437044; doi:10.1038/s41598-022-19306-1)
Supplement: Supplementary file 1 — Supplementary Information 1. [file 41598_2022_19306_MOESM1_ESM.docx]

# **Supplementary information**

**Electrospun P3HT/PVDF-HFP Semiconductive Nanofibers for Triboelectric Nanogenerators**

Meng-Fang Lin*, Gang-Wei Zhang, Jia-Xian Li, Xin-Xain Wu, Yu-Ching Huang *

*Department of Materials Engineering, Ming Chi University of Technology, New Taipei City, Taiwan*


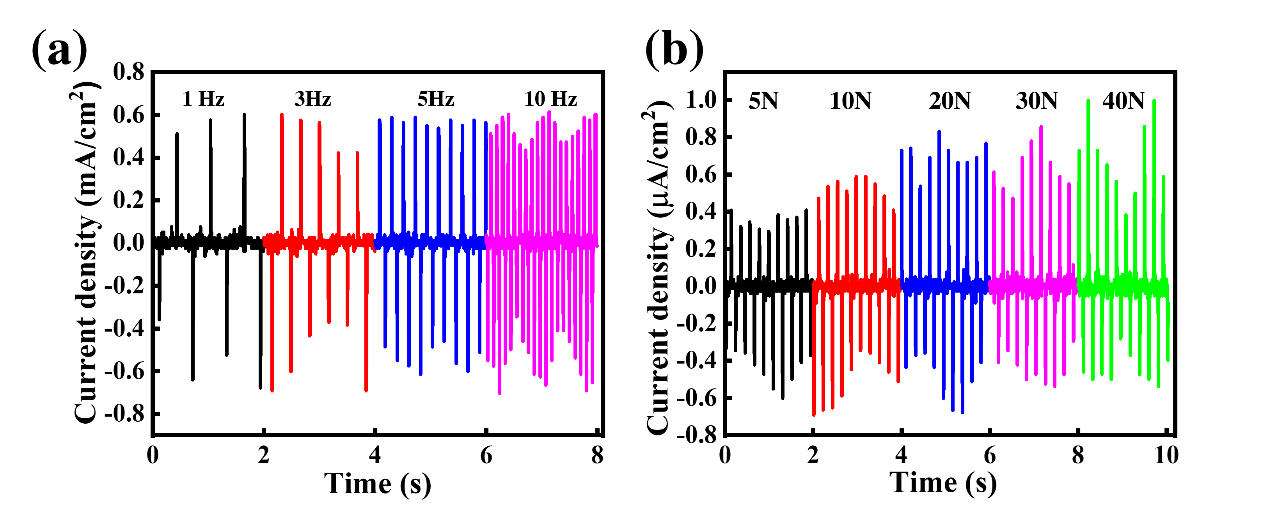


**Fig. S1** Electrical output current density of the P3HT/PVDF-HFP nanofibers measured with respect to (a) frequency and (b) mechanical force.


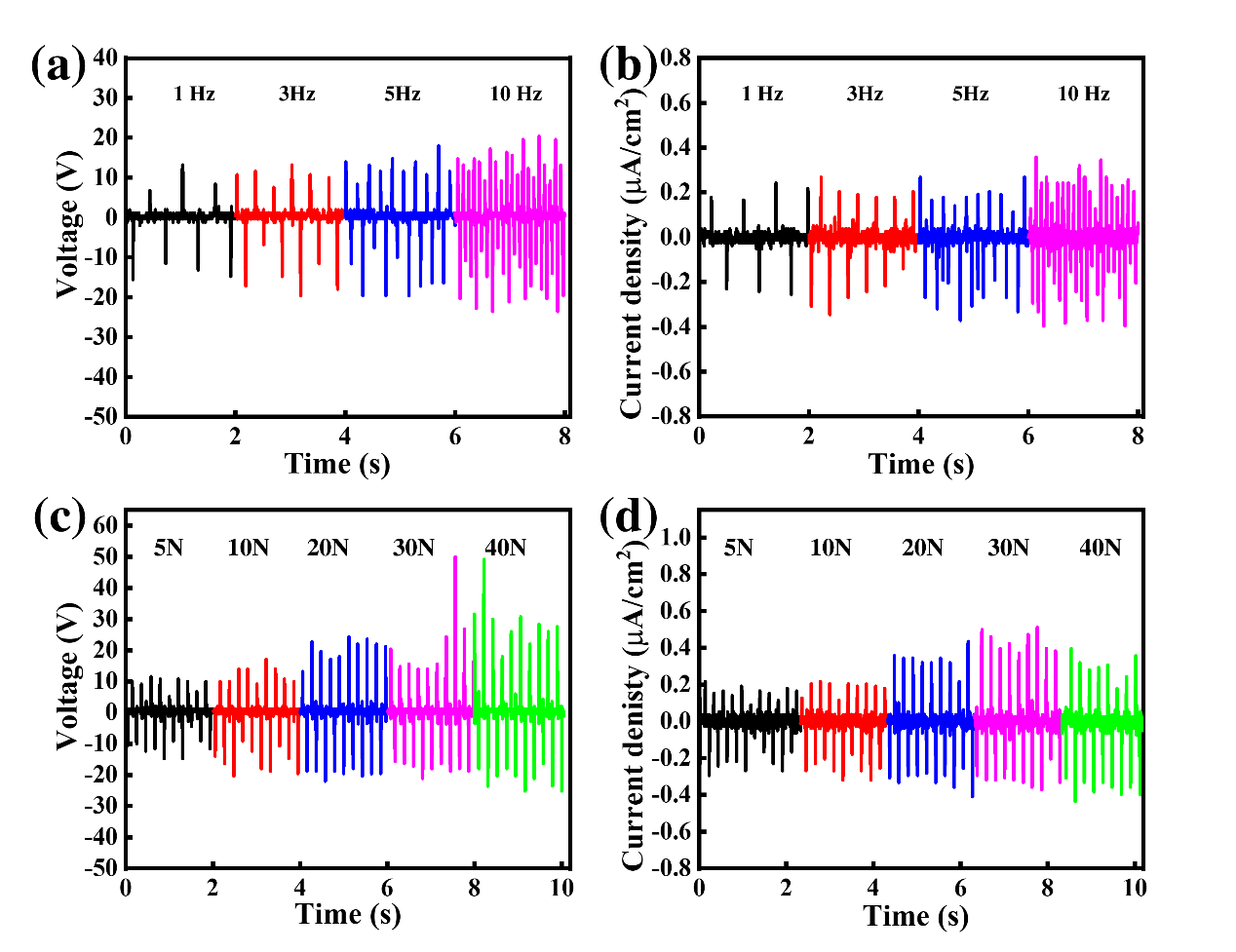


**Fig. S2** (a, c) Output voltages and (b, d) current densities of the TENG composed of electrospun PVDF-HFP nanofibers, measured (a, b) under the same mechanical force (10 N) at frequencies (*f*) of 1, 3, 5, and 10 Hz and (c, d) under the same frequency (5 Hz) at mechanical forces of 5, 10, 20, 30, and 40 N.


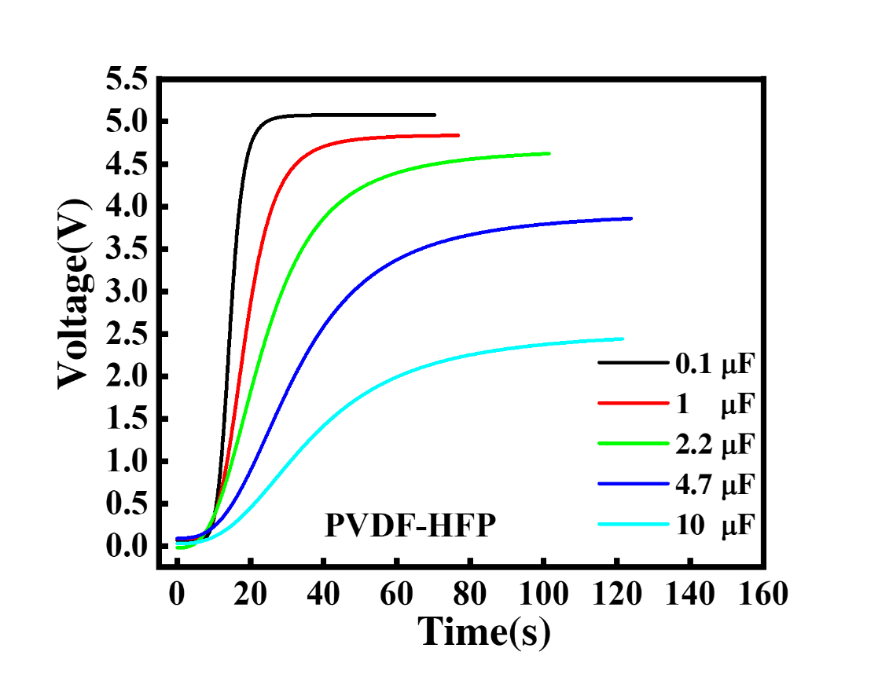


**Fig. S3** (a) Capacitor charging of the PVDF-HFP nanofiber TENG at various capacitances.


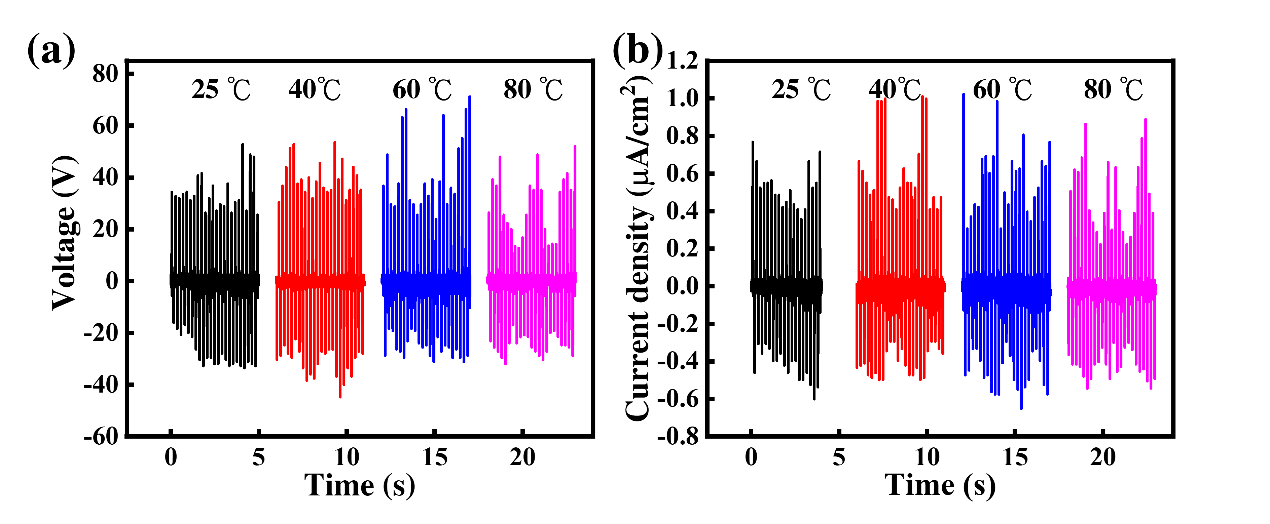


**Fig. S4** (a) Voltage and (b) current outputs of the P3HT/PVDF-HFP nanofiber TENG, measured at various temperatures.

**Movie** **S1.** LED bulb array and a digital watch could be powered by the TENG containing the P3HT/PVDF-HFP nanofiber mats
